# Volumetric changes associated with immediate implant placement and Bio-Oss collagen

**DOI:** 10.6026/973206300214469

**Published:** 2025-12-15

**Authors:** Harsh Ranjan, Shamna Alathankandy Vaniyanveetti, Srilekha Pulivarthi, Sachin Gendlalji fulbe, Muzammil Moin Ahmed, Manaswini Draksharapu, Md Kafeel Ahmed

**Affiliations:** 1 Department of Dental and Surgery, Oral and Maxillofacial Surgery, Consultant at Narayan Hospital and Ambika Hospital, Patna, Bihar, India; 2 Department of Periodontology and Implantology, Associate Dentist, Markham and Associates, Reading, Berkshire, England, UK; 3 Department of Oral Medicine and Radiology, MNR Dental College and Hospital, Sangareddy, Telangana, India; 4 Department of Periodontics, Triveni Institute of Dental Sciences, Hospital and Research Centre, Bodri, Chhattisgarh, India; 5 Department of Public Health, College of Applied Medical Sciences, Qassim University, Buraydah, Saudi Arabia; 6 Department of Prosthodontics Crown Bridge and Implantology, Mamata Dental College, Khammam, Telangana, India; 7 Department of Periodontology and Implantology, MNR Dental College and Hospital, Sangareddy, Hyderabad, Telangana, India

**Keywords:** Immediate implant placement, Bio-Oss collagen, volumetric analysis, bone resorption, CBCT, ridge preservation

## Abstract

Volumetric changes associated with immediate implant placement and Bio-Oss collagen is observed. Hence, Thirty patients requiring single-tooth extraction and immediate implant placement in the anterior maxilla were randomly allocated into two groups: Group A (n=15) received immediate implants with Bio-Oss Collagen filling the gap and Group B (n=15) received immediate implants without grafting. Cone-beam computed tomography (CBCT) scans were obtained immediately post-operatively and at six months. Volumetric measurements were performed using three-dimensional image analysis software. Statistical analysis included paired and independent t-tests. Bio-Oss Collagen significantly reduced volumetric bone resorption following immediate implant placement, demonstrating superior preservation of peri-implant hard tissue dimensions compared to non-grafted sites.

## Background:

Immediate implant placement, defined as implant insertion into the extraction socket during the same surgical procedure, has gained widespread acceptance as a treatment option that reduces treatment time and the number of surgical interventions [1]. This approach offers several advantages including preservation of the alveolar bone architecture, maintenance of soft tissue contours and improved patient satisfaction [2, 3]. However, despite these benefits, dimensional changes of the alveolar ridge following tooth extraction and immediate implant placement remain inevitable due to physiological bone remodeling processes [4]. Studies have demonstrated that significant horizontal and vertical bone resorption occurs within the first six months following tooth extraction, with horizontal dimensional changes ranging from 29% to 63% and vertical changes from 11% to 22% [5, 6]. These dimensional alterations can compromise aesthetic outcomes, particularly in the anterior maxillary region where tissue stability is paramount for achieving natural-appearing restorations [7]. The presence of a gap between the implant surface and the socket wall, commonly known as the "jumping distance," has been identified as a critical factor influencing the extent of bone remodeling [8].

Various biomaterials have been proposed to fill this peri-implant defect and minimize dimensional changes, including autogenous bone, allografts, xenografts and alloplastic materials [9, 10]. Among these, Bio-Oss Collagen, a deproteinized bovine bone mineral combined with 10% porcine collagen, has demonstrated favorable osteoconductive properties and volume stability [11, 12]. The collagen component provides cohesiveness and facilitates handling, while the mineral phase serves as a scaffold for new bone formation [13]. Although several studies have investigated dimensional changes following immediate implant placement with various grafting materials, most have relied on two-dimensional linear measurements [14, 15]. Three-dimensional volumetric analysis using cone-beam computed tomography (CBCT) provides more comprehensive and accurate assessment of bone changes, eliminating errors associated with conventional two-dimensional measurements [16]. However, limited data exist regarding volumetric changes specifically associated with Bio-Oss Collagen in immediate implant protocols. Furthermore, the clinical significance of volumetric preservation in relation to aesthetic outcomes and long-term implant success requires further investigation [17]. Understanding the three-dimensional behavior of peri-implant tissues when Bio-Oss Collagen is utilized could provide valuable information for treatment planning and patient selection. Therefore, it is of interest to quantitatively evaluate three-dimensional volumetric changes in the peri-implant region following immediate implant placement with and without Bio-Oss Collagen grafting material over a six-month healing period using CBCT analysis.

## Materials and Methods:

## Study design:

This prospective, randomized, controlled clinical trial was conducted at the Department of Oral and Maxillofacial Surgery between January 2022 and December 2023.

## Sample size calculation:

Sample size calculation was performed using G*Power software version 3.1.9.4, based on a previous pilot study. Assuming an effect size of 0.8, alpha error of 0.05 and power of 80%, a minimum of 13 patients per group was required. Accounting for potential dropouts, 15 patients per group (total n=30) were recruited.

## Patient selection:

## Inclusion criteria:

[1] Age between 18 and 65 years

[2] Requirement for single-tooth extraction in the anterior maxillary region (canine to canine)

[3] Adequate bone quantity for primary implant stability (minimum 35 Ncm)

[4] Intact buccal bone plate confirmed clinically and radiographically

[5] Good oral hygiene (plaque index <20%)

[6] Systemically healthy patients (ASA I or II)

## Exclusion criteria:

[1] Active infection at the surgical site

[2] Inadequate bone volume for primary stability

[3] Smoking >10 cigarettes per day

[4] Uncontrolled diabetes mellitus (HbA1c >7%)

[5] History of bisphosphonate therapy

[6] Pregnancy or lactation

[7] Previous radiation therapy to the head and neck region

[8] Acute periapical pathology extending beyond 5 mm from the apex

## Randomization and blinding:

Patients were randomly assigned to one of two groups using computer-generated randomization. Allocation was concealed using sealed opaque envelopes opened only during surgery. The radiographic evaluator was blinded to group allocation throughout the study.

## Surgical protocol:

All surgical procedures were performed by a single experienced surgeon. Patients received prophylactic antibiotics (amoxicillin 2g) one hour pre-operatively. Under local anesthesia (articaine 4% with epinephrine 1:100,000), teeth were carefully extracted using periotomes and elevators, minimizing trauma to the surrounding bone. Socket walls were thoroughly debrided and examined for integrity. Group A (Test Group, n=15): Immediate implant placement with Bio-Oss Collagen grafting. Tapered implants (Straumann BLT, Switzerland) were placed 3-4 mm apical to the buccal bone crest. The gap between implant and socket wall was filled with Bio-Oss Collagen (Geistlich Pharma AG, Switzerland). A collagen membrane was placed over the socket opening and tension-free primary closure was achieved. Group B (Control Group, n=15): Immediate implant placement without grafting. The same implant system and surgical protocol were followed, but no grafting material was placed in the peri-implant gap. Natural blood clot formation was allowed. All patients received post-operative instructions and medications (amoxicillin 500 mg three times daily for seven days, ibuprofen 400 mg as needed). Sutures were removed after 10-14 days.

## CBCT imaging and volumetric analysis:

CBCT scans (Planmeca ProMax 3D Mid, Finland) were obtained immediately post-operatively (T0) and at six months (T6) using standardized parameters (90 kV, 10 mA, 0.2 mm voxel size, field of view 8×8 cm). Patients were positioned using a customized bite registration to ensure reproducible head positioning. DICOM data were imported into three-dimensional analysis software (Mimics Innovation Suite 23.0, Materialise, Belgium). A standardized region of interest (ROI) was defined as the area extending 3 mm mesially, distally and apically from the implant platform and encompassing the entire buccal bone plate. Semi-automatic segmentation was performed using threshold values of 226-3071 Hounsfield Units for mineralized tissue. Volumetric measurements (cm^3^) were calculated automatically by the software. Linear measurements were also recorded at three levels: 1 mm apical to implant platform (coronal), mid-implant level (middle) and 1 mm from implant apex (apical) for horizontal dimensional changes.

## Statistical analysis:

Data were analyzed using SPSS version 26.0 (IBM Corporation, USA). Normality was assessed using the Shapiro-Wilk test. Descriptive statistics were calculated as mean ± standard deviation (SD) for continuous variables. Paired t-tests compared within-group changes between T0 and T6. Independent t-tests compared between-group differences. The significance level was set at p<0.05. Intra-examiner reliability was assessed by repeating measurements on 10 randomly selected scans after two weeks, calculating intraclass correlation coefficients (ICC).

## Results:

All 30 patients (17 females, 13 males) completed the six-month follow-up period with no dropouts. Mean age was 34.2 ± 8.6 years in Group A and 36.8 ± 9.3 years in Group B (p=0.43). All implants achieved primary stability exceeding 35 Ncm (Group A: 42.3 ± 5.2 Ncm; Group B: 41.1 ± 4.8 Ncm; p=0.52). No implant failures or complications occurred during the observation period, resulting in a 100% survival rate in both groups. Intra-examiner reliability demonstrated excellent agreement (ICC = 0.96; 95% CI: 0.91-0.98). Table 1 (see PDF) presents the volumetric measurements and changes over the six-month period. At baseline (T0), mean peri-implant bone volume was comparable between groups (Group A: 1.46 ± 0.12 cm^3^; Group B: 1.47 ± 0.15 cm^3^; p=0.84). At six months (T6), Group A demonstrated significantly greater volume preservation (1.28 ± 0.10 cm^3^) compared to Group B (1.05 ± 0.12 cm^3^; p<0.001). The absolute volumetric reduction was significantly lower in Group A (0.18 ± 0.06 cm^3^) compared to Group B (0.42 ± 0.09 cm^3^; p<0.001). When expressed as percentage, Group A showed 12.3 ± 3.7% volumetric loss versus 28.6 ± 5.4% in Group B (p<0.001), representing a 57% reduction in volumetric bone loss when Bio-Oss Collagen was utilized. Horizontal dimensional changes at three vertical levels are presented in Table 2 (see PDF). At the coronal level (1 mm apical to implant platform), Group A exhibited significantly less horizontal resorption (1.2 ± 0.4 mm) compared to Group B (2.8 ± 0.7 mm; p<0.001). At the mid-implant level, the difference remained statistically significant (Group A: 0.8 ± 0.3 mm; Group B: 2.1 ± 0.6 mm; p<0.001). At the apical level, both groups showed minimal changes with less pronounced differences (Group A: 0.3 ± 0.2 mm; Group B: 0.7 ± 0.3 mm; p<0.001). The greatest dimensional changes occurred at the coronal level in both groups, with Group B demonstrating more than twice the horizontal resorption compared to Group A at all measured levels. Table 3 (see PDF) illustrates the distribution of patients according to the magnitude of volumetric bone loss. In Group A, 80% of patients experienced less than 15% volumetric loss, with only 20% showing 15-20% loss. No patient in Group A exceeded 20% volumetric reduction. Conversely, in Group B, only 13.3% maintained less than 20% loss, while 40% experienced 25-30% loss and 33.4% showed more than 30% volumetric bone loss.

## Discussion:

The present study evaluated three-dimensional volumetric changes following immediate implant placement with and without Bio-Oss Collagen grafting over a six-month healing period. The findings demonstrated that Bio-Oss Collagen significantly reduced peri-implant bone resorption, with the grafted group showing 12.3% volumetric loss compared to 28.6% in the non-grafted group. These results support the hypothesis that filling the peri-implant gap with xenogeneic grafting material effectively preserves bone volume during the critical early healing phase. The observed volumetric bone loss in the non-grafted group aligns with previous literature reporting substantial dimensional changes following immediate implant placement [18, 19]. Ferrus and colleagues reported mean horizontal resorption of 1.0-4.5 mm in non-grafted immediate implant sites, which is consistent with our findings of 2.8 mm at the coronal level [20]. The physiological remodeling process, combined with the presence of a jumping distance between implants and socket wall, contributes to these dimensional alterations [21]. The mechanism by which Bio-Oss Collagen preserves bone volume involves several biological processes. The deproteinized bovine bone mineral provides a three-dimensional scaffold that maintains space and prevents soft tissue collapse into the defect [22]. The osteoconductive properties facilitate migration of osteogenic cells and vessel ingrowth, promoting new bone formation [23]. Additionally, the slow resorption rate of Bio-Oss ensures long-term volume stability, as particles become integrated within newly formed bone [24, 25]. The collagen component enhances handling properties and provides a matrix for cell adhesion and proliferation [26]. Our volumetric analysis using CBCT represents advancement over traditional two-dimensional measurements, which can underestimate actual bone changes due to projection errors and landmark identification difficulties [27]. Three-dimensional assessment provides comprehensive information about the entire peri-implant region, capturing changes that might be missed by linear measurements alone [28]. Previous studies utilizing similar three-dimensional methodologies have reported comparable findings, with grafted sites showing superior dimensional stability [29, 30]. The distribution analysis revealed that 80% of patients in the Bio-Oss Collagen group maintained volumetric loss below 15%, whereas only 13.3% of non-grafted patients achieved this level of preservation. This clinically relevant finding suggests that Bio-Oss Collagen provides predictable outcomes for the majority of patients, which is crucial for treatment planning, particularly in aesthetically demanding situations [31]. The greater variability observed in the non-grafted group may reflect individual differences in healing capacity and bone remodeling patterns [32].

Horizontal dimensional changes showed a gradient pattern, with greatest resorption occurring at the coronal level and decreasing apically. This corresponds to the thinner buccal bone plate typically present at the crestal region, which is more susceptible to remodeling [33]. The preservation of horizontal dimensions is particularly important for maintaining proper implant positioning relative to the final restoration and ensuring adequate soft tissue support [34]. The 1.6 mm difference in horizontal resorption at the platform level between groups has significant clinical implications for achieving optimal emergence profiles and avoiding aesthetic complications such as recession [35]. The 100% implant survival rate in both groups confirms that immediate implant placement is a predictable procedure when appropriate patient selection and surgical protocols are followed [36]. The achievement of adequate primary stability (>35 Ncm) in all cases was essential for successful osseointegration [37]. While survival rates were equivalent between groups, the superior dimensional preservation in the grafted group suggests better long-term prognosis for maintaining peri-implant tissues and aesthetic outcomes [38]. Several limitations of this study should be acknowledged. The six-month observation period, while adequate for assessing early remodeling, does not provide information about long-term stability. Future studies should include extended follow-up to evaluate volumetric changes over several years [39]. Additionally, this study focused exclusively on the anterior maxilla; results may differ in other anatomical locations with varying bone quality and aesthetic demands [40]. The sample size, although adequate for statistical power, was relatively small and multicenter studies with larger populations would strengthen the evidence base. Furthermore, this study did not assess the histological composition of preserved volume, which would provide valuable information about the proportion of newly formed bone versus residual graft material [41]. Correlation between volumetric preservation and aesthetic outcomes, including soft tissue parameters and patient satisfaction, represents an important area for future investigation [42].

## Conclusion:

Bio-Oss Collagen significantly reduces volumetric bone resorption following immediate implant placement in the anterior maxilla. At six months, grafted sites showed 12.3% volumetric loss compared to 28.6% in non-grafted sites, representing a clinically significant difference. Horizontal dimensional changes were similarly reduced at all vertical levels when Bio-Oss Collagen was utilized. The superior dimensional preservation achieved with Bio-Oss Collagen suggests its routine use should be considered in immediate implant protocols, particularly in aesthetically critical regions where tissue stability is paramount for optimal outcomes.
